# Leukocyte Telomere Length-Related rs621559 and rs398652 Genetic Variants Influence Risk of HBV-Related Hepatocellular Carcinoma

**DOI:** 10.1371/journal.pone.0110863

**Published:** 2014-11-03

**Authors:** Wenting Pan, Guangxia Cheng, Huaixin Xing, Juan Shi, Chao Lu, Jinyu Wei, Lichao Li, Changchun Zhou, Qipeng Yuan, Liqing Zhou, Ming Yang

**Affiliations:** 1 State Key Laboratory of Chemical Resource Engineering, Beijing Laboratory of Biomedical Materials, College of Life Science and Technology, Beijing University of Chemical Technology, Beijing, China; 2 Clinical Laboratory, Jinan Infectious Disease Hospital, Shandong University, Jinan, Shandong Province, China; 3 Department of Anesthesiology, Shandong Cancer Hospital, Shandong Academy of Medical Sciences, Jinan, Shandong Province, China; 4 Clinical Laboratory, Shandong Cancer Hospital, Shandong Academy of Medical Sciences, Jinan, Shandong Province, China; 5 Department of Radiation Oncology, Huaian No. 2 Hospital, Huaian, Jiangsu Province, China; Ohio State University Medical Center, United States of America

## Abstract

Recent genome-wide association studies (GWAS) have identified eleven leukocyte telomere length (LTL)-related single nucleotide polymorphisms (SNPs). Since LTL has been associated with risk of many malignancies, LTL-related SNPs may contribute to cancer susceptibility. To test this hypothesis in hepatitis B virus (HBV)-related hepatocellular carcinoma (HCC), we genotyped these eleven LTL-related SNPs in a case-control set including 1186 HBV-related HCC cases, 508 chronic HBV carriers and 1308 healthy controls at the discovery stage. The associations of HCC risk with these SNPs were further confirmed in an independent case-control set. We found that 1p34.2 rs621559 and 14q21 rs398652 were significantly associated with HBV-related HCC risk (both *P*<0.005 after Bonferroni corrections). There was no significant difference of either rs621559 or rs398652 genotypes between chronic HBV carriers and healthy controls, demonstrating that the association was not due to predisposition to HBV infection. In the pooled analyses (1806 HBV-related HCC cases and 1954 controls), we observed a decreased HCC risk, 0.72-times, associated with the 1p34.2 rs621559 AA genotype compared to the GG genotype (*P* = 1.6×10^−6^). Additionally, there was an increased HCC risk, 1.27-fold, associated with the rs398652 GG genotype (*P* = 3.3×10^−6^). A statistical joint effect between the rs621559 GG and rs398652 GG genotypes may exist in elevating risk of HBV-related HCC. We show, for the first time, that rs398652 and rs621559 might be marker genetic variants for risk of HBV-related HCC in the Chinese population.

## Introduction

Human telomeres, consisting of “TTAGGG” short repetitive sequences, locate at the ends of chromosomes [1], [2]. The core function of telomeres is to protect chromosomes' integrity, prevent chromosomal instability, and avoid the activation of DNA-damage responses [1], [2]. Because of the “end replication problem”, telomeres would shorten after each DNA replication and each cell division in normal cells. When telomeres shrink to be tremendously short, these normal cells would go through cell-cycle arrest, apoptosis or senescence [1]. Nevertheless, in cancer cells, telomerase can synthesize telomere repeats *de novo* and overcome telomere attrition [1]–[4]. Therefore, malignant cells with over-expressed telomerase can unlimitedly grow *in vitro* and *in vivo* [1]. However, in normal human leukocytes without or with little telomerase activity, telomere length shortens with age at a rate of 20∼40 base pairs (bp) per year [5], [6]. Leukocyte telomere length (LTL) has been associated with risk to developing many malignancies [7]–[18], highlighting the predictive role of LTL in carcinogenesis.

It has been reported that LTL is genetically heritable, with heritability ranging from 44% to 80% [19]–[21]. Recently, several genome-wide association studies (GWAS) identified eleven single nucleotide polymorphisms (SNPs) which are associated with LTL in different ethnic populations [9], [22]–[25]. For example, Gu J et al. found that four SNPs (rs398652 on 14q21, rs621559 on 1p34.2, rs6028466 on 20q11.22 and rs654128 on 6q22.1) were associated with LTL in Caucasian populations (pooled *P*<10^−5^). In a large case–control study, they observed that subjects with the variant allele of rs398652 has a significantly reduced risk of bladder cancer [Odds ratio (OR) = 0.81; 95% Confidence interval (CI) = 0.67–0.97; *P* = 0.025], consistent with the correlation of this variant allele with longer telomeres. In a previous study, we also investigated whether these four genetic variants are associated with LTL and risk of esophageal squamous cell carcinoma (ESCC) in Chinese populations [26]. After measuring LTL of 550 healthy individuals, we verified that both rs621559 and rs398652 genetic variants are significantly associated with LTL. On the basis of analyzing 1550 ESCC patients and frequency-matched 1620 controls from 4 medical centers in China, we found that the rs621559 AA genotype is associated with a decreased risk of ESCC, 0.71-times, compared to the rs621559 GG genotype (*P* = 5.9×10^−6^). We also detected a moderately increased OR for ESCC that was associated with the 14q21 rs398652 G allele (*P* = 6.5×10^−4^). The aforementioned results indicate that these LTL-related SNPs identified by GWAS [9], [22]–[25], might contribute to cancer susceptibility.

As the third third-most cause of cancer mortality in the world, hepatocellular carcinoma (HCC) shows unbalanced distribution, with the highest morbidity in Asia and Sub-Saharan Africa [27], [28]. Notably, China alone accounts for approximately half of all HCC patients [27]. Epidemiological evidences indicate that chronic infections with the hepatitis B or C viruses (HBV or HCV), exposure to dietary aflatoxin B as well as alcohol abuse are major risk factors. In China, HBV infection is prevalent and particular important, due to its coherent distribution with HCC [27], [28]. However, only a fraction of HBV chronic carriers developed HCC, suggesting that genetic makeup may also contribute to development of HBV-related HCC [27], [28]. The existence of genetic polymorphisms influencing the development of HBV-related HCC has also been proved by several GWAS on HBV-related HCC in eastern Asians [29]–[32].

Taken together, we hypothesized that the LTL-related SNPs identified by GWAS [9], [22]–[25], may influence HBV-related HCC risk in Chinese populations. To test this hypothesis, we conducted a two-stage case–control study of HBV-related HCC from different regions of China.

## Materials and Methods

### Study case-control sets

This study consisted of two case-control sets: (a) Shandong set (Discovery set): 1186 patients with HBV-related HCC, sex- and age-matched (±5 years) 508 chronic HBV carriers as well as sex- and age-matched 1308 healthy controls. HBV-related HCC patients and chronic HBV carriers were recruited between June 2009 and October 2012 at Shandong Cancer Hospital, Shandong Academy of Medical Sciences (Jinan, Shandong Province, China). Control subjects were randomly selected from a pool of 4500 individuals from a community cancer-screening program for cancer early detection conducted in Jinan city during the same time period as the patients were collected. Part of the case-control set has been reported previously [29]. (b) Jiangsu set (Validation set): 560 HBV-related HCC patients from Huaian No. 2 Hospital (Huaian, Jiangsu Province, China) and sex- and age-matched 566 controls. Patients were consecutively recruited between January 2009 and September 2012 at Huaian No. 2 Hospital. Controls were cancer-free individuals selected from a community cancer-screening program (3000 individuals) for early detection of cancer conducted in Huaian city during the same time period as the patients were collected. Part of the case-control set has been reported previously [33], [34]. The diagnosis of all patients was confirmed by a pathological examination combined with positive imaging (magnetic resonance imaging and/or computerized tomography). All participants were negative for antibodies to HCV, hepatitis D virus or HIV since we excluded all participants with HCV, hepatitis D virus or HIV infection. Chronic HBV carriers and HBV-related HCC cases were defined as individuals with the following serological parameters [HBsAg(+) for>6 months, anti-HBc(+), and anti-HBs(-)]. Healthy controls are ones without HBV infection. Individuals who smoked one cigarette per day for over 1 year were considered as smokers. Subjects were considered as alcohol drinkers, if they drank at least once per week. All subjects were ethnic Han Chinese. At recruitment, the written informed consent was obtained from each subject. This study was approved by the institutional Review Boards of Shandong Cancer Hospital and Huaian No. 2 Hospital.

### Polymorphism genotyping

During the discovery stage, all eleven LTL-related SNPs (rs398652 ref.9, rs621559 ref.9, rs6028466 ref.9, rs654128 ref.9, rs12696304 ref.22, rs3772190 ref.23, rs4452212 ref.23, rs4387287 ref.23, rs2162440 ref.24, rs7235755 ref.24, and rs16847897 ref.25) were firstly analyzed in the Shandong case-control set (Discovery set) by the MassArray system (Sequenom Inc., San Diego, California, USA). A 15% blind, random sample of study subjects was genotyped in duplicates and the reproducibility was 100%. To reduce experimental costs, we developed a PCR-based restriction fragment length polymorphism (RFLP) method to determine genotypes of two SNPs (rs398652 and rs621559) in the validation case-control set (Jiangsu set). In PCR-RFLP genotyping, the primers used for amplifying DNA segments containing the rs398652 and rs621559 site (mismatch bases are underlined) were 5′-GAAACTAATTCTTGCTGTCTCGA-3′/5′-CCTTCACTCCTGCTCATTTCC-3′, or 5′-AGTGTTCCTTTGCCTCATCTA-3′/5′-ACTCTGTGGAACAGTTGGGTA-3′. Restriction enzymes *Xho*I and *Rsa*I (New England Biolabs) were used to distinguish the rs398652 A>G and rs621559 G>A genotypes. A 15% random sample was reciprocally tested by different person, and the reproducibility was 99.5%.

### Statistical analyses

Pearson's χ^2^ test was used to examine the differences in demographic variables, smoking status, drinking status, and genotype distributions of eleven LTL-related polymorphisms between patients and controls. The associations between genotypes of these genetic variants and HBV-related HCC risk were estimated by ORs and their 95% CIs computed by logistic regression models. All ORs were adjusted for age, sex, smoking or drinking status, where it was appropriate. A *P* value of less than 0.05 was used as the criterion of statistical significance, and all statistical tests were two-sided. All analyses were performed using SPSS 16.0 (SPSS Inc.).

## Results

No statistically significant differences were found between HBV-related HCC patients, chronic HBV carriers and healthy controls for Shandong set and Jiangsu set in terms of median age and sex distribution (*P*>0.05). This indicates that the frequency matching was adequate (Table 1). However, smokers were over-represented among patients compared with controls in both Shandong and Jiangsu case-control sets (*P*<0.05). In addition, we observed that there are more subjects who drink alcohol among cases than those among controls in both case-control sets (*P*<0.001).

**Table 1:** Distribution of selected characteristics among HBV-related HCC cases, chronic HBV carriers and controls.

Variable	Shandong set (Discovery set)					Jiangsu set (Validation set)		
	HCC cases	Chronic HBV carriers	Healthy controls	*P* a	*P* b	HCC cases	Healthy controls	*P* b
	No. (%)	No. (%)	No. (%)			No. (%)	No. (%)	
	1186	508	1308			620	646	
Age (year)				0.626	0.457			0.860
≤57	627(52.9)	262(51.6)	672(51.4)			315(50.8)	325(50.3)	
>57	559(47.1)	246(48.4)	636(48.6)			305(49.2)	321(49.7)	
Sex				0.249	0.969			0.348
Male	1018(85.8)	425(83.7)	1122(85.8)			531(85.6)	541(83.7)	
Female	168(14.2)	83(16.3)	186(14.2)			89(14.4)	105(16.3)	
Smoking status				0.104	<0.001			<0.001
No	468(39.5)	222(43.7)	687(52.5)			214(34.5)	376(58.2)	
Yes	718(60.5)	286(56.3)	621(47.5)			406(65.5)	270(41.8)	
Drinking status				0.133	<0.001			<0.001
No	410(34.6)	195(38.4)	607(46.4)			156(25.2)	355(55.0)	
Yes	776(65.4)	313(61.6)	701(53.6)			464(74.8)	291(45.0)	

Note: HBV, hepatitis B virus; HCC, hepatocellular carcinoma.

a Two-sided χ^2^ test, HCC cases vs. chronic HBV carriers.

b Two-sided χ^2^ test, HCC cases vs. healthy controls.

Firstly, unconditional logistic regression analysis was utilized to calculate associations between genotypes of eleven LTL-related SNPs (rs398652, rs621559, rs6028466, rs654128, rs12696304, rs3772190, rs4452212, rs4387287, rs2162440, rs7235755, and rs16847897) and HBV-related HCC risk in Shandong discovery set (Table 2). Among eleven SNPs, rs6028466 showed no frequency in Chinese and was excluded in further analyses. In Shandong set consisting of 1186 patients with HBV-related HCC, 508 chronic HBV carriers as well as 1308 healthy controls, we found that two common SNPs (14q21 rs398652 and 1p34.2 rs621559) were significantly associated with HBV-related HCC risk (both *P*<0.005 after Bonferroni corrections; HCC cases vs. healthy controls). However, none of these LTL-related SNPs were associated with chronic HBV infection when different genotypes were compared between chronic HBV carriers and healthy controls. Therefore, we believe that the positive association between rs398652 or rs621559 SNPs and HBV-related HCC was not due to predisposition to HBV infection. Interestingly, these two SNPs were associated with LTL in healthy Chinese subjects in our previous study [26]. The rs621559 A allele was a protective allele; subjects with the AG or AA genotype had an OR of 0.72 (95% CI = 0.61–0.85, *P* = 1.2×10^−4^) or 0.74 (95% CI = 0.63–0.87, *P* = 2.2×10^−4^) for developing HBV-related HCC, respectively, compared with subjects with the GG genotype (Trend test, *P* = 1.2×10^−6^) (Table 3). The odds of having the rs398652 GG genotype in patients was 1.26 (95% CI = 1.12–1.42, *P* = 1.3×10^−4^) compared with the AA genotype. A significantly increased cancer risk was also associated with the rs398652 AG genotype (OR = 1.41, 95% CI = 1.19–1.68, *P* = 9.5×10^−5^) (Trend test, *P* = 0.026) (Table 4). All ORs were adjusted for sex, age, smoking and drinking status.

**Table 2:** Associations between candidate leukocyte telomere length-related genetic variants and risk of HBV-related HCC in Shandong case-control set (Discovery set).

No.	Literatures	rs ID	Base change	MAF^a^	Genotype (1186 HCC cases, 508 Chronic HBV carriers and 1308 healthy controls)						
					Common^b^	Heterozygous^b^	Rare^b^	OR^c^ (95% CI)	*P*	OR^d^ (95% CI)	*P*
1	Gu et al.	rs621559	G>A	0.290	58.4/50.9/50.0	36.0/41.7/42.0	5.6/7.4/8.0	0.76(0.66–0.86)	1.5×10^−5^	0.97(0.82–1.14)	0.690
2	Gu et al.	rs398652^e^	A>G	0.360	34.9/43.1/41.9	48.1/43.8/44.2	17.0/13.1/13.9	1.30(1.16–1.46)	6.0×10^−6^	0.99(0.85–1.15)	0.880
3	Gu et al.	rs6028466	G>A	0	0/0/0	0/0/0	0/0/0	N.A.	N.A.	N.A.	N.A.
4	Gu et al.	rs654128	G>T	0.017	96.4/96.5/96.6	3.6/3.5/3.4	0/0/0	0.93(0.59–1.45)	0.724	0.97(0.54–1.74)	0.901
5	Codd et al.	rs12696304	C>G	0.325	46.0/45.4/45.6	43.7/43.7/43.8	10.3/10.9/10.6	0.98(0.87–1.11)	0.757	0.99(0.86–1.16)	0.915
6	Levy et al.	rs3772190	T>C	0.426	31.5/31.4/31.0	52.9/52.8/52.7	15.6/15.8/16.3	0.97(0.87–1.09)	0.613	0.98(0.84–1.13)	0.754
7	Levy et al.	rs4452212	A>G	0.020	96.5/96.2/96.0	3.4/3.8/4.0	0.1/0/0	0.91(0.59–1.39)	0.652	0.94(0.53–1.64)	0.818
8	Levy et al.	rs4387287	A>C	0.158	70.3/70.7/71.0	27.3/26.8/26.4	2.4/2.5/2.6	0.98(0.84–1.15)	0.822	0.99(0.81–1.21)	0.907
9	Mangino et al.	rs2162440	A>G	0.217	61.5/61.0/61.5	33.7/33.9/33.7	4.8/5.1/4.8	1.00(0.87–1.15)	0.999	1.02(0.85–1.22)	0.826
10	Mangino et al.	rs7235755	A>G	0.217	61.5/61.0/61.5	33.6/33.9/33.7	4.9/5.1/4.8	1.00(0.87–1.15)	0.999	1.02(0.85–1.22)	0.826
11	Prescott et al.	rs16847897	G>C	0.399	35.4/35.8/36.6	48.5/47.1/46.9	16.1/17.1/16.5	0.98(0.88–1.10)	0.776	0.97(0.84–1.13)	0.723

Note: HBV, hepatitis B virus; HCC, hepatocellular carcinoma; MAF, minor allele frequency; N.A., not available.

a MAF in controls.

b % of HCC case/% of chronic HBV carriers/% of control.

c Allelic OR calculated by logistic regression (HCC cases vs. healthy controls).

d Allelic OR calculated by logistic regression (Chronic HBV carriers vs. healthy controls).

e There are 1184 HCC cases, 502 Chronic HBV carriers and 1c02 healthy controls which have been successfully genotyped.

**Table 3:** Genotype frequencies of the 1p34.2 rs621559 G>A polymorphism among HCC cases, chronic HBV carriers and healthy controls and their association with HBV-related HCC risk.

Studies	Genotypes	HCC cases, No. (%)	Chronic HBV carriers, No. (%)	Healthy controls, No. (%)	OR^a^ (95% CI)	*P* a	OR^b^ (95% CI)	*P* b
		*n* = 1186	*n* = 508	*n* = 1308				
Shandong set	AA	693(58.4)	259(50.9)	646(49.4)	1.00 (Reference)		1.00 (Reference)	
	AG	429(36.2)	212(41.7)	551(42.1)	0.72(0.61–0.85)	1.2×10^−4^	0.93(0.71–1.16)	0.873
	GG	64(5.4)	38(7.4)	111(8.5)	0.74(0.63–0.87)	2.2×10^−4^	0.88(0.63–1.22)	0.519
*P*_trend_ c						1.2×10^−6^		0.450
		*n* = 620		*n* = 646				
Jiangsu set	AA	373(60.2)	N.A.	327(50.6)	1.00 (Reference)			
	AG	217(35.0)	N.A.	260(40.2)	0.73(0.57–0.94)	0.015	N.A.	N.A.
	GG	30(4.8)	N.A.	59(9.1)	0.69(0.54–0.88)	0.003	N.A.	N.A.
*P*_trend_ c						8.2×10^−5^		
		*n* = 1806		*n* = 1954				
Pooled	AA	1066(59.0)	N.A.	973(49.8)	1.00 (Reference)			
	AG	646(35.8)	N.A.	811(41.5)	0.73(0.64–0.84)	9.5×10^−6^	N.A.	N.A.
	GG	94(5.2)	N.A.	170(8.7)	0.72(0.63–0.82)	1.6×10^−6^	N.A.	N.A.
*P*_trend_ c						4.2×10^−10^		

Note: HBV, hepatitis B virus; HCC, hepatocellular carcinoma; OR, odds ratio; CI, confidence interval; N.A., not available.

a HCC case vs. healthy controls, data were calculated by logistic regression with adjustment for age, sex, smoking and drinking.

b Chronic HBV carriers vs. healthy controls, data were calculated by logistic regression with adjustment for age, sex, smoking and drinking.

c Test for trend of odds was two-sided and based on likelihood ratio test assuming a multiplicative model.

**Table 4:** Genotype frequencies of the 14q21 rs398652 A>G polymorphism among HCC cases, chronic HBV carriers and healthy controls and their association with HBV-related HCC risk.

Studies	Genotypes	HCC cases, No. (%)	Chronic HBV carriers, No. (%)	Healthy controls, No. (%)	OR^a^ (95% CI)	*P* a	OR^b^ (95% CI)	*P* b
		*n* = 1184	*n* = 502	*n* = 1302				
Shandong set	AA	413(34.9)	216(43.1)	570(43.8)	1.00 (Reference)		1.00 (Reference)	
	AG	570(48.1)	220(43.8)	558(42.8)	1.41(1.19–1.68)	9.5×10^−5^	0.99(0.85–1.26)	0.814
	GG	201(17.0)	66(13.1)	174(13.4)	1.26(1.12–1.42)	1.3×10^−4^	1.00(0.77–1.35)	0.927
*P*_trend_ c						0.026		0.878
		*n* = 620		*n* = 645				
Jiangsu set	AA	233(37.5)	N.A.	307(47.6)	1.00 (Reference)			
	AG	299(48.3)	N.A.	275(42.6)	1.27(0.99–1.64)	0.064	N.A.	N.A.
	GG	88(14.2)	N.A.	63(9.8)	1.23(1.01–1.50)	0.039	N.A.	N.A.
*P*_trend_ c						1.2×10^−4^		
		*n* = 1804		*n* = 1947				
Pooled	AA	646(35.8)	N.A.	877(45.0)	1.00 (Reference)			
	AG	869(48.2)	N.A.	833(42.8)	1.40(1.21–1.62)	4.1×10^−6^	N.A.	N.A.
	GG	289(16.0)	N.A.	237(12.2)	1.27(1.15–1.41)	3.3×10^−6^	N.A.	N.A.
*P*_trend_ c						6.5×10^−9^		

Note: HBV, hepatitis B virus; HCC, hepatocellular carcinoma; OR, odds ratio; CI, confidence interval; N.A., not available.

a HCC case vs. healthy controls, data were calculated by logistic regression with adjustment for age, sex, smoking and drinking.

b Chronic HBV carriers vs. healthy controls, data were calculated by logistic regression with adjustment for age, sex, smoking and drinking.

c Test for trend of odds was two-sided and based on likelihood ratio test assuming a multiplicative model.

The associations of HBV-related HCC risk with these two SNPs were further verified in an independent case-control set. Genotyping results showed that these two SNPs were both significantly associated with HBV-related HCC risk in Jiangsu Chinese population (Table 3 and 4). Carriers of rs621559 AA genotype showed significantly and consistently decreased risks to develop HBV-related HCC compared with rs621559 GG carriers (OR = 0.69, 95% CI = 0.54–0.88, *P* = 0.003) (Table 3). Significantly reduced HBV-related HCC risk was observed among rs621559 AG carriers compared to individuals with GG rs621559 genotype in Jiangsu case-control set (OR = 0.73, 95% CI = 0.57–0.94, *P* = 0.015) (Table 3). Individuals with rs398652 GG genotype also had significantly increased HBV-related HCC risk compared with those with rs398652 AA genotype in the validation set (OR = 1.23, 95% CI = 1.01–1.50, *P* = 0.039) (Table 4). Logistic regression analyses also revealed that individuals with rs398652 AG genotype were associated with an increased risk, 1.27-fold, to develop HBV-related HCC in the validation set (*P* = 0.064) (Table 4).

In the pooled analyses, we found that the rs621559 AG or AA genotype carriers had a decreased risk, 0.73-times or 0.72-times, to develop HBV-related HCC compared to the GG genotype carriers (95% CI = 0.64–0.84, *P* = 9.5×10^−6^ or 95% CI = 0.63–0.82, *P* = 1.6×10^−6^) (Trend test, *P* = 4.2×10^−10^) (Table 3). Similarly, rs398652 AG or GG genotype was also significantly associated with HBV-related HCC risk (OR = 1.40, 95% CI = 1.21–1.62, *P* = 4.1×10^−6^; OR = 1.27, 95% CI = 1.15–1.41, *P* = 3.3×10^−6^) compared to the AA genotype (Trend test, *P* = 6.5×10^−9^) (Table 4). We also examined whether there are gene-environment interaction between 14q21 rs398652 or 1p34.2 rs621559 polymorphism and age, sex, smoking or drinking history, but the results were negative.

Because 14q21 rs398652 or 1p34.2 rs621559 polymorphism alone was respectively associated with risk of HBV-related HCC, we further examined whether there was a statistically joint effect between rs621559 and rs398652 genotypes on HCC risk (Table 5). We found that patients who carried the rs621559 GG genotype were also more likely to carry the rs398652 GG genotype than controls (9.6% versus 5.9%; *P* = 3.1×10^−8^). The presence of the rs621559 GG genotype or rs398652 GG genotype alone was associated with an increased risk of HBV-related HCC (OR = 2.52, 95% CI = 1.57–4.05, *P* = 1.2×10^−4^; or OR = 3.34, 95% CI = 2.07–5.37, *P* = 7.0×10^−7^) compared with the absence of such a genotype. However, the presence of both rs621559 GG and rs398652 GG genotypes was associated with an even higher risk of HBV-related HCC (OR = 4.16, 95% CI = 2.51–6.90, *P* = 3.1×10^−8^) compared with the lack of both genotypes. These results indicate that a statistical joint effect between the rs621559 GG and rs398652 GG genotypes may exist in elevating risk of HBV-related HCC.

**Table 5:** Risk of HBV-related HCC associated with 1p34.2 rs621559 genotypes by 14q21 rs398652 genotypes.

Genotypes					
rs621559 (1p34.2)	rs398652 (14q21)	Patients, No. (%)	Controls, No. (%)	OR^a^ (95% CI)	*P*
AA	AA	27(1.5)	76(3.9)	1.00 (Reference)	
AA	AG	51(2.8)	73(3.7)	2.11(1.18–3.79)	0.012
AA	GG	16(0.9)	19(1.0)	3.34(2.07–5.37)	7.0×10^−7^
AG	AA	247(13.7)	379(19.5)	1.84(1.14–2.98)	0.012
AG	AG	298(16.5)	328(16.8)	2.88(1.82–4.56)	5.8×10^−6^
AG	GG	100(5.5)	103(5.3)	2.68(1.58–4.52)	2.4×10^−4^
GG	AA	372(20.6)	422(21.7)	2.52(1.57–4.05)	1.2×10^−4^
GG	AG	520(28.8)	432(22.2)	3.32(2.08–5.30)	5.2×10^−7^
GG	GG	173(9.6)	115(5.9)	4.16(2.51–6.90)	3.1×10^−8^

Note: HBV, hepatitis B virus; HCC, hepatocellular carcinoma.

a Data were calculated by logistic regression, adjusted for sex, age, smoking, and drinking.

## Discussion

It has been found that short LTL was associated with increased susceptibilities of several malignancies [7]–[17]. Therefore, it is rational that genetic variants, which are associated with shortened LTL, may contribute to increased cancer risk. In previous GWA studies, eleven SNPs have been identified with significant association with shortened LTL [9], [22]–[25]. In the current study, we investigated if these SNPs may influence the risk of HBV-related HCC in Chinese. After genotyping 1806 HBV-related HCC cases, 508 chronic HBV carriers and frequency-matched 1954 healthy controls, we observed a decreased HCC risk, 0.72-times, associated with the 1p34.2 rs621559 AA genotype compared with the rs621559 GG genotype (*P* = 1.6×10^−6^). Additionally, there was an increased risk for HBV-related HCC, 1.27-fold, associated with the 14q21 rs398652 GG genotype in Chinese (*P* = 3.3×10^−6^).

In a previous study, Gu J et al. showed that subjects with the variant allele of rs398652 (associated with longer telomeres) has a significantly reduced risk of bladder cancer [9]. In addition, we found that both rs621559 and rs398652 genetic variants have obvious impact on LTL and ESCC susceptibility in Chinese [26]. In line with these findings, our current study also confirmed that only rs621559 and rs398652 influence risk of HBV-related HCC among all eleven GWAS-identified LTL-related SNPs. These findings demonstrate that rs621559 and rs398652 might be common genetic components in intensifying risk of different malignancies.

Another possible explanation of this inconsistence is that rs621559 and rs398652 SNPs may also contribute to HCC risk through influence function and/or expression of genes in a trans-regulation way. rs398652 locates about 60 kb from the *PELI2* gene, encoding the pellino2 protein which plays a pivotal role in inflammatory response and production of cytokines. Interestingly, it has been shown that chronic inflammation is associated with up to 25% of malignancies and also accelerates telomere attrition [35]–[37]. Therefore, it is possible that chronic inflammation caused by HBV infection can partly elucidates associations between rs398652 and telomere length as well as HCC risk. For rs621559, there is no report on its host, the *WDR65* gene, shows its involvement in either telomere maintenance or carcinogenesis.

Our results are obtained from two independent case-control analyses derived from different Han Chinese populations. Having relative large sample sizes, significantly increased/decreased ORs with small *P* values, these results are unlikely to be attributable to selection bias or unknown confounding factors. The fact that genotype frequencies of all SNPs fit Hardy–Weinberg equilibrium and are identical in the three populations further supports the randomness of our subject selection. Therefore, further studies of HCC and other cancers would be helpful to verify our results.

In all, to the best of our knowledge, this is the first study to reveal that the LTL-related 1p34.2 rs621559 and 14q21 rs398652 polymorphisms influence development of HBV-related HCC.

## References

[1] Blackburn EH (2001) Switching and signaling at the telomere. Cell 106: 661–673. 11572773 10.1016/s0092-8674(01)00492-5

[2] Brown WR (1989) Molecular cloning of human telomeres in yeast. Nature 338: 774–776. 2541342 10.1038/338774a0

[3] Shampay J, Szostak JW, Blackburn EH (1984) DNA sequences of telomeres maintained in yeast. Nature 310: 154–157. 6330571 10.1038/310154a0

[4] Blackburn EH, Greider CW, Szostak JW (2006) Telomeres and telomerase: the path from maize, Tetrahymena and yeast to human cancer and aging. Nat Med 12: 1133–1138. 17024208 10.1038/nm1006-1133

[5] Valdes AM, Andrew T, Gardner JP, Kimura M, Oelsner E, et al (2005) Obesity, cigarette smoking, and telomere length in women. Lancet 366: 662–664. 16112303 10.1016/S0140-6736(05)66630-5

[6] Aviv A (2006) Telomeres and human somatic fitness. J Gerontol A Biol Sci Med Sci 61: 871–873. 16912107 10.1093/gerona/61.8.871

[7] Xing J, Ajani JA, Chen M, Izzo J, Lin J, et al (2009) Constitutive short telomere length of chromosome 17p and 12q but not 11q and 2p is associated with an increased risk for esophageal cancer. Cancer Prev Res (Phila) 2: 459–465. 19401529 10.1158/1940-6207.CAPR-08-0227 PMC2701666

[8] Blasco MA (2005) Telomeres and human disease: ageing, cancer and beyond. Nat Rev Genet 6: 611–622. 16136653 10.1038/nrg1656

[9] Gu J, Chen M, Shete S, Amos CI, Kamat A, et al (2011) A genome-wide association study identifies a locus on chromosome 14q21 as a predictor of leukocyte telomere length and as a marker of susceptibility for bladder cancer. Cancer Prev Res (Phila) 4: 514–521. 21460395 10.1158/1940-6207.CAPR-11-0063 PMC3076128

[10] McGrath M, Wong JY, Michaud D, Hunter DJ, De Vivo I (2007) Telomere length, cigarette smoking, and bladder cancer risk in men and women. Cancer Epidemiol Biomarkers Prev 16: 815–819. 17416776 10.1158/1055-9965.EPI-06-0961

[11] Shao L, Wood CG, Zhang D, Tannir NM, Matin S, et al (2007) Telomere dysfunction in peripheral lymphocytes as a potential predisposition factor for renal cancer. J Urol 178: 1492–1496. 17707063 10.1016/j.juro.2007.05.112

[12] Willeit P, Willeit J, Mayr A, Weger S, Oberhollenzer F, et al (2010) Telomere length and risk of incident cancer and cancer mortality. JAMA 304: 69–75. 20606151 10.1001/jama.2010.897

[13] Wu X, Amos CI, Zhu Y, Zhao H, Grossman BH, et al (2003) Telomere dysfunction: a potential cancer predisposition factor. J Natl Cancer Inst 95: 1211–1218. 12928346 10.1093/jnci/djg011

[14] Zheng YL, Zhou X, Loffredo CA, Shields PG, Sun B (2011) Telomere deficiencies on chromosomes 9p, 15p, 15q and Xp: potential biomarkers for breast cancer risk. Hum Mol Genet 20: 378–386. 20956286 10.1093/hmg/ddq461 PMC3005901

[15] Mirabello L, Garcia-Closas M, Cawthon R, Lissowska J, Brinton LA, et al (2010) Leukocyte telomere length in a population-based case-control study of ovarian cancer: a pilot study. Cancer Causes Control 21: 77–82. 19784860 10.1007/s10552-009-9436-6 PMC3130499

[16] Broberg K, Björk J, Paulsson K, Höglund M, Albin M (2005) Constitutional short telomeres are strong genetic susceptibility markers for bladder cancer. Carcinogenesis 26: 1263–1271. 15746160 10.1093/carcin/bgi063

[17] Jones AM, Beggs AD, Carvajal-Carmona L, Farrington S, Tenesa A, et al (2012) TERC polymorphisms are associated both with susceptibility to colorectal cancer and with longer telomeres. Gut 61: 248–254. 21708826 10.1136/gut.2011.239772 PMC3245900

[18] Liu J, Yang Y, Zhang H, Zhao S, Liu H, et al (2011) Longer leukocyte telomere length predicts increased risk of hepatitis B virus-related hepatocellular carcinoma: a case-control analysis. Cancer 117: 4247–4256. 21387275 10.1002/cncr.26015

[19] Njajou OT, Cawthon RM, Damcott CM, Wu SH, Ott S, et al (2007) Telomere length is paternally inherited and is associated with parental lifespan. Proc Natl Acad Sci U S A 104: 12135–12139. 17623782 10.1073/pnas.0702703104 PMC1924539

[20] Vasa-Nicotera M, Brouilette S, Mangino M, Thompson JR, Braund P, et al (2005) Mapping of a major locus that determines telomere length in humans. Am J Hum Genet 76: 147–151. 15520935 10.1086/426734 PMC1196417

[21] Slagboom PE, Droog S, Boomsma DI (1994) Genetic determination of telomere size in humans: a twin study of three age groups. Am J Hum Genet 55: 876–882. 7977349 PMC1918314

[22] Codd V, Mangino M, van der Harst P, Braund PS, Kaiser M, et al (2010) Common variants near TERC are associated with mean telomere length. Nat Genet 42: 197–199. 20139977 10.1038/ng.532 PMC3773906

[23] Levy D, Neuhausen SL, Hunt SC, Kimura M, Hwang SJ, et al (2010) Genome-wide association identifies OBFC1 as a locus involved in human leukocyte telomere biology. Proc Natl Acad Sci U S A 107: 9293–9298. 20421499 10.1073/pnas.0911494107 PMC2889047

[24] Mangino M, Richards JB, Soranzo N, Zhai G, Aviv A, et al (2009) A genome-wide association study identifies a novel locus on chromosome 18q12.2 influencing white cell telomere length. J Med Genet 46: 451–454. 19359265 10.1136/jmg.2008.064956 PMC2696823

[25] Prescott J, Kraft P, Chasman DI, Savage SA, Mirabello L, et al (2011) Genome-wide association study of relative telomere length. PLoS One 6: e19635. 21573004 10.1371/journal.pone.0019635 PMC3091863

[26] Shi J, Sun F, Peng L, Li B, Liu L, et al (2013) Leukocyte telomere length-related genetic variants in 1p34.2 and 14q21 loci contribute to the risk of esophageal squamous cell carcinoma. Int J Cancer 132: 2799–2807. 23180556 10.1002/ijc.27959

[27] Parkin DM, Bray F, Ferlay J, Pisani P (2005) Global cancer statistics, 2002. CA Cancer J Clin 55: 74–108. 15761078 10.3322/canjclin.55.2.74

[28] El-Serag HB, Rudolph KL (2007) Hepatocellular carcinoma: epidemiology and molecular carcinogenesis. Gastroenterology 132: 2557–2576. 17570226 10.1053/j.gastro.2007.04.061

[29] Jiang DK, Sun J, Cao G, Liu Y, Lin D, et al (2013) Genetic variants in STAT4 and HLA-DQ genes confer risk of hepatitis B virus-related hepatocellular carcinoma. Nat Genet 45: 72–75. 23242368 10.1038/ng.2483 PMC4105840

[30] Li S, Qian J, Yang Y, Zhao W, Dai J, et al (2012) GWAS identifies novel susceptibility loci on 6p21.32 and 21q21.3 for hepatocellular carcinoma in chronic hepatitis B virus carriers. PLoS Genet 8: e1002791. 22807686 10.1371/journal.pgen.1002791 PMC3395595

[31] Chan KY, Wong CM, Kwan JS, Lee JM, Cheung KW, et al (2011) Genome-wide association study of hepatocellular carcinoma in Southern Chinese patients with chronic hepatitis B virus infection. PLoS One 6: e28798. 22174901 10.1371/journal.pone.0028798 PMC3234276

[32] Zhang H, Zhai Y, Hu Z, Wu C, Qian J, et al (2010) Genome-wide association study identifies 1p36.22 as a new susceptibility locus for hepatocellular carcinoma in chronic hepatitis B virus carriers. Nat Genet 42: 755–758. 20676096 10.1038/ng.638

[33] Liu L, Zhou C, Zhou L, Peng L, Li D, et al (2012) Functional FEN1 genetic variants contribute to risk of hepatocellular carcinoma, esophageal cancer, gastric cancer and colorectal cancer. Carcinogenesis 33: 119–123. 22072618 10.1093/carcin/bgr250

[34] Zhou L, Zhang X, Chen X, Liu L, Lu C, et al (2012) GC Glu416Asp and Thr420Lys polymorphisms contribute to gastrointestinal cancer susceptibility in a Chinese population. Int J Clin Exp Med 5: 72–79. 22328951 PMC3272689

[35] von Zglinicki T (2002) Oxidative stress shortens telomeres. Trends Biochem Sci 27: 339–344. 12114022 10.1016/s0968-0004(02)02110-2

[36] Houben JM, Mercken EM, Ketelslegers HB, Bast A, Wouters EF, et al (2009) Telomere shortening in chronic obstructive pulmonary disease. Respir Med 103: 230–236. 18945604 10.1016/j.rmed.2008.09.003

[37] Masi S, Salpea KD, Li K, Parkar M, Nibali L, et al (2011) Oxidative stress, chronic inflammation, and telomere length in patients with periodontitis. Free Radic Biol Med 50: 730–735. 21195167 10.1016/j.freeradbiomed.2010.12.031

